# Effect of K^+^ Diffusion on Hydration of Magnesium Potassium Phosphate Cement with Different Mg/P Ratios: Experiments and Molecular Dynamics Simulation Calculations

**DOI:** 10.3390/ma17051151

**Published:** 2024-03-01

**Authors:** Difei Leng, Qiuyan Fu, Yunlu Ge, Chenhao He, Yang Lv, Xiangguo Li

**Affiliations:** State Key Laboratory of Silicate Materials for Architectures, Wuhan University of Technology, No. 122 at Luoshi Road, Wuhan 430070, China; lengdifei@126.com (D.L.); fuqiuyan01@126.com (Q.F.); geyunlu01@126.com (Y.G.); hechenhao01@126.com (C.H.)

**Keywords:** magnesium potassium phosphate cement, K-struvite, newberyite, diffusion kinetics of K^+^

## Abstract

Magnesium potassium phosphate cement (MKPC) is formed on the basis of acid–base reaction between dead burnt MgO and KH_2_PO_4_ in aqueous solution with K-struvite as the main cementitious phase. Due to the unique characteristics of these cements, they are suitable for special applications, especially the immobilization of radioactive metal cations and road repair projects at low temperature. However, there are few articles about the hydration mechanism of MKPC. In this study, the types, proportions and formation mechanism of MKPC crystalline phases under different magnesium to phosphorus (Mg/P) ratios were studied by means of AAS, ICP-OES, SEM, EDS and XRD refinement methods. Corresponding MD simulation works were used to explain the hydration mechanism. This study highlights the fact that crystalline phases distribution of MKPC could be adjusted and controlled by different Mg/P ratios for the design of the MKPC, and the key factor is the kinetic of K^+^.

## 1. Introduction

Phosphate materials have been used as dental fillings since the 19th century [1]. Subsequently, new phosphate materials were further developed as fire retardant materials and emergency road repair materials in low-temperature environments [2]. In the field of orthopedics, they are promising materials for accelerating bone regeneration, including calcium phosphate, magnesium ammonium phosphate and the latest potassium magnesium phosphate [3]. Moreover, they can be used for the stabilization/solidification of radioactive metal cations because of their excellent immobilization effects, great encapsulation properties and excellent resistance to acid attack and high temperature [4,5,6,7,8,9,10,11]. Among various kinds of phosphate cements, magnesium potassium phosphate cement (MKPC) has drawn a great interest in research. Although mass replacement of OPC is unlikely as a kind of Mg-based cement, MKPC would generate less CO_2_ emissions [12]. MKPC is formed by the acid–base reaction between dead burnt MgO and KH_2_PO_4_, as shown in Equation (1):

MgO + KH_2_PO_4_ + 5H_2_O→MgKPO_4_·6H_2_O (1)

In recent years, practicability [13,14,15,16], new retarders [17,18], durability [19,20] and effects of new admixtures [18,21,22,23,24,25,26,27,28,29] were focuses of research on MKPC. However, the hydration mechanism of MKPC has always been one of the key issues. Some researchers have offered their insights. At the beginning of this century, E Soudee et al. firstly studied the solidification reaction mechanism of magnesium phosphate cement, pointing out that MgO continuously adsorbed two water molecules at the step position on its surface to form Mg(H_2_O)_6_^2+^, and then gradually reacted with PO_4_^3−^ and NH_4_^+^ in aqueous solution to bond into cement phase [30]. Their work highlighted the important role of surfaces in the reaction of magnesium phosphate cement. Wagh proposed a basic mechanism that Mg(H_2_O)_6_^2+^ appears first, and these ions become dense K-struvite gel around unreacted MgO [31]. Mathieu Le Rouzic et al. believe that newberyite (MgHPO_4_·3H_2_O) is an important transition product whose formation is sensitive to pH value [32]. Besides newberyite, cattite (Mg_3_(PO_4_)_2_·22H_2_O) [33], phosphorrösslerite (MgHPO_4_·7H_2_O) [34] and Mg_2_KH(PO_4_)_2_·15H_2_O [35,36] are also transition products during early hydration. However, there is little literature about how much of the MgO is converted to the effective cementing material K-struvite phase rather than other crystalline phases, especially the effect of the Mg/P ratios [34] on the hydration process and hydration products. In addition, little attention has been paid to the byproducts newberyite and brucite [Mg(OH)_2_]. Although there is little mention in the scientific literature of expansion and cracking of MKPC [34], several experimental reports have described its expansion and cracking [37]. Newberyite and brucite are considered as potential risk factors for expansion and cracking of MKPC [32].

However, most of the existing studies focused on the dissolution stage of MgO, while the cementation process of Mg^2+^, K^+^ and H_2_PO_4_^−^ is rarely studied. With the dissolution of MgO, ions in the solution undergoing chemical reactions will obviously affect the performance of MKPC. In particular, it is noted that Mg^2+^ is always produced on the surface of the solid MgO, and there is a certain physical distance between Mg^2+^ and K^+^ in solution. K^+^ diffuses to the surface of MgO in aqueous solution and meets Mg^2+^ to finally react. The diffusion kinetics of K^+^ would play a decisive role in the hydration reaction because if K^+^ diffusion is too low, resulting in a lack of K^+^, the system would generate a high ratio of newberyite (MgHPO_4_·3H_2_O, no K^+^ in this phase), which does not require K^+^ rather than the target product K-struvite (MgKPO_4_·6H_2_O, K^+^ exists in in this phase). More deadly, newberyite has a volume effect, which lead to a potential risk of cracking in the MKPC system [32]. This is particularly critical for MKPC systems used to immobilize radioactive nuclides, as even small cracks could expand the exposed area of the solidified matrix, greatly increasing its risk of releasing radioactive nuclides into the environment.

Under this background, the main objective of this work is to reveal the formation mechanism of K-struvite and newberyite under different Mg/P ratios in magnesium phosphate cements, particularly the degree of dead burnt MgO converting to the other crystalline phases. This work paid special attention to the influence of K^+^ diffusion kinetics on MKPC systems. A workable MKPC system would be designed by adjusting to an appropriate Mg/P ratio. Molecular dynamics (MD) simulations were used to understand the hydration mechanism of MKPC at the microscopic level. 

## 2. Methodology

### 2.1. Experimental Method

**Figure 1:**
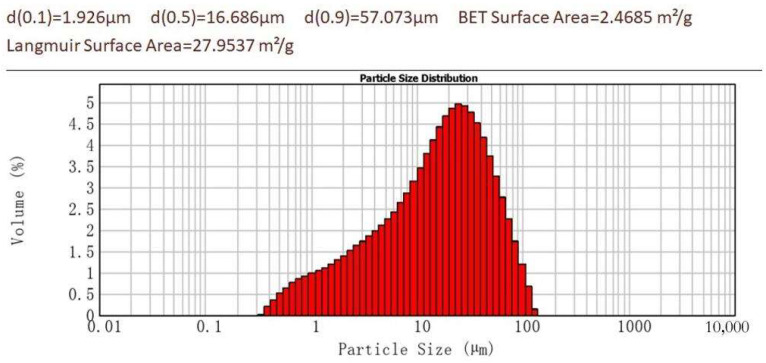
PSD, BET surface area and Langmuir Surface Area of dead burnt MgO.

To understand the formation mechanism of K-struvite and newberyite under different Mg/P ratios in MKPC, dead burnt MgO (from Qiaoxu Magnesium Material Factory, Yingkou, China), analytical reagent KH_2_PO_4_ (from Shanghai Aladdin, Shanghai, China) and ultra-pure water were used to generate cement crystal phases. Usually, some supplementary materials such as fly ash (FA), silica fume (SF) [38] and retarder borax are introduced to MKPC systems. In this study, these supplementary materials are avoided for a cleaner hydration mechanism analysis. The MgO was ground and selected by a 200-mesh sieve. Chemical compositions of dead burnt MgO were analyzed by the X-ray fluorescence (XRF) method (shown in Table 1). The particle size and specific surface area of dead burnt MgO were analyzed by laser particle size analyzer and BET analysis, respectively (shown in Figure 1). The citric acid reactivity time of the dead burnt MgO was 7200s. The mixtures with different Mg/P molar ratios of 1:1, 3:1, 5:1 and 7:1 were designated and labeled as MP1, MP3, MP5 and MP7, respectively (shown in Table 2). In this work, no retarder was used. The water-to-total-solids (w/s) was 10. 

Here, the reason for choosing w/s as 10 needs to be explained. Firstly, the author needs to point out that MKPC system is a kind of fast-setting cement, and it is difficult to obtain filtrate liquid using a conventional 0.4 w/s value, and studying hydration mechanism requires the experimental determination of K^+^ concentration and phosphorus-containing anion concentration in the filtrate liquid. Secondly, it is usually appropriate to use a large w/s value when studying the mechanism of MKPC. For example, MgCl_2_ solution, instead of solid MgO, was used directly to obtain the MKPC system [32], and the hydration mechanism of MKPC was studied. Thirdly, even if the liquid/powder value is large, it still has the sandwich cake structure of [MgO particle-water-MgO particle] on the microscopic level. The local microscopic liquid/powder in fact would not be far away from a 0.4 w/s value, even we used a 10 w/s value. Finally, the results of MD simulation with a 0.4 w/s value are consistent with AAS, ICP-OES and other experimental results with a 10 w/s value. Therefore, it is appropriate to study the hydration mechanism of MKPC with a 10 w/s value.

The specific experimental operation was to stir and dissolve 17.01 g KH_2_PO_4_ in 220.49 g, 321.25 g, 422.01 g and 522.77 g deionized water (MP1, MP3, MP5 and MP7, respectively), and then add 5.89 g, 17.66 g, 29.43 g and 41.20 g dead-burnt MgO (MP1, MP3, MP5 and MP7, respectively) to the solution with stirring. The pH value of the system was measured from the time once MgO was added. During the curing time, it was sealed with a teflon membrane and placed in a 298.15 K calorstat. After each measurement, deionized water was added to supplement the amount of evaporated water, after which it was sealed with the teflon membrane and returned to the calorstat. The whole process lasted 21 days, and these samples were separated by negative pressure suction filter device to obtain the leachate and solid samples. Solid samples were dried to a constant weight in a 323.15 K drying oven for subsequent testing.

Concentrations of K^+^ in leachate were analyzed by atomic absorption spectrometry instrument (CONTRAA-700 from Analytik Jena AG, Jena, Germany) and concentrations of P by Inductively Coupled Plasma-Optical Emission Spectroscopy (Prodigy 7 from Hudson, NH, USA). The test standard obeys GB/T 15337-2008. The ICP-OES test is direct-reading. The precision from XRF is ±0.0005 mg/L. The crystal phases were analyzed by X-ray diffractometry instrument (Empyrean from Malvern Panalytical, Almelo, The Netherlands, 10° < 2θ < 65°, 1.83°/min). XRD was recorded on an Empyrean instrument using Cu K_α_ radiation (40 kV, 40 mA). Rigaku Smart Lab (https://www.rigaku.com/products/xrd/smartlab, accessed on 19 February 2024) was used to perform the Rietveld analysis. The elemental distribution and morphology of the products were analyzed by X-ray photo electron spectroscopy (ESCALAB 250Xi from Thermo Fisher Scientific Co., Waltham, MA, USA) and scanning electron microscopy (JSM-IT300 from JEOL Ltd., Akishima, Japan). The acceleration voltage is 20 kV. The quantification method is standardless quantitative analysis. The PSD, BET surface area and Langmuir Surface Area of dead burnt MgO were tested by automatic surface area and porosity analyzer (ASAP 2020 from Malvern Instruments Limited, Britain). The test mode is general purpose under a nitrogen atmosphere. The PSD-BET test is direct-reading.

### 2.2. Computational Method

This work was based on traditional molecular dynamics (MD) simulation calculations. All models were simulated with an NVT process during 20 ns (using Velocity-rescale thermostat with every 0.2 ps) after an NPT process during 80 ps by GROMACS [39,40,41,42,43,44]. The force field file was adopted by ‘gromos54a7.ff’ and the non-bond parameters of K^+^ and Mg^2+^ by ‘kbff20.ff’. The non-bond parameters of H_2_PO_4_^−^, HPO_4_^2−^ and MgO were obtained by Sobtop. In these simulations, Ewald sum method was deployed to cope with long-range electrostatics under 1.2 nm and tail corrections for long-range Lennard-Jones interactions. We adopted a timestep of 1 fs. The simulated temperature was 320 K.

In this work, MD simulations of MKPC system were carried out to investigate the effect of K^+^ diffusion on hydration process under different Mg/P ratios. The initial K^+^ were located at 25–45 Å, away from the MgO solid base surface (4.21 Å). The initial Mg^2+^ were located at 5–25 Å. The water–solid ratio (w/s) of each model was set at 0.4. The ratios of HPO_4_^2−^ to H_2_PO_4_^−^ were obtained from Equation (2) by the pH measured by experiment (seen in Section 3.1) and the pK_a_ of H_2_PO_4_^−^:

pH-pK_a_ = lg ([H_2_PO_4_^−^]/[HPO_4_^2−^]) (2)

**Figure 2:**
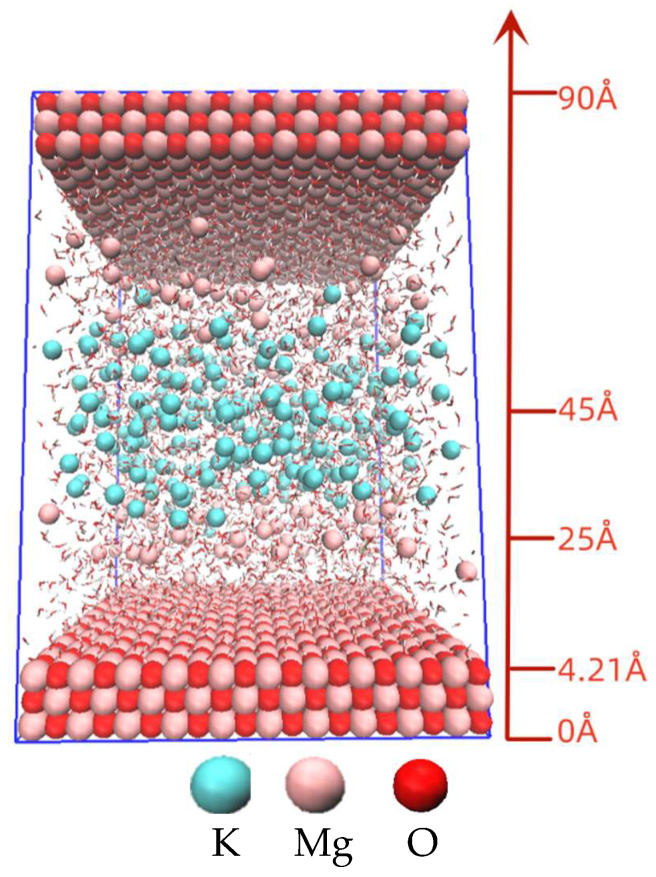
Initial distribution of ions in MD models.

The initial MD models were established by Packmol [45], shown in Table 3 and Figure 2:

## 3. Results and Discussion

### 3.1. pH Value

**Figure 3:**
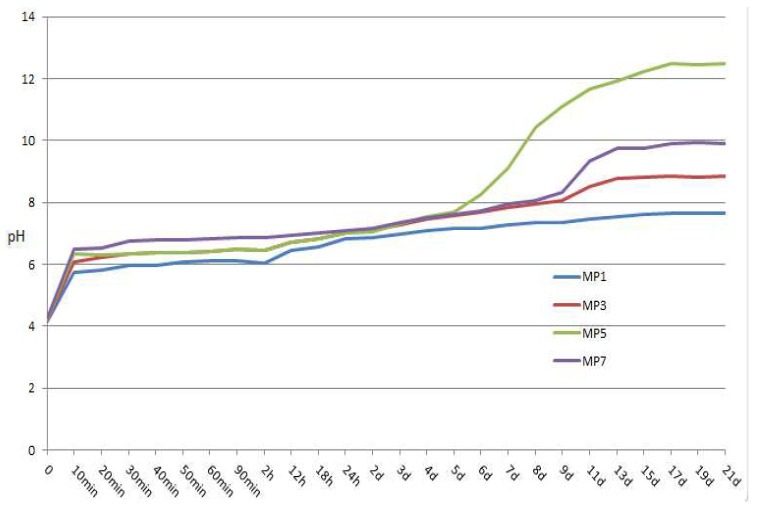
pH of solution of MKPC systems.

It is well known that pH has extensive and profound effects on the acid–base reaction in solution. Le Rouzic et al. [32] pointed out that newberyite is the only crystalline substance in the solution of MKPC system at pH < 7. It deserves further study here. Figure 3 shows the pH of solutions of MKPC samples under different Mg/P up to 21 d. The end time of K-struvite formation depends on reactivity of dead burnt MgO, usually up to 2 h. This work believes the end time was up to 5 d as a 7200 s citric acid reactivity time. Overall, the pH value of the solution increases gradually with the processing hydration, which is consistent with the literature description [46]. Before 5 days, the general pH value is MP7 > MP5 > MP3 > MP1. After 5 days, the general pH value is MP5 > MP7 > MP3 > MP1. This study believes that pH value is one of the important factors affecting the type and population of hydration products in the system, not only because a suitable pH is a prerequisite for the formation of newberyite and K-struvite but also because when pH > 7, pH value controls the dissolving of MgO in the solution. The generation rate of K-struvite determines the thickness of the K-struvite layer attached to the surface of MgO, and this K-struvite is converted from MgO. 

**Figure 4:**
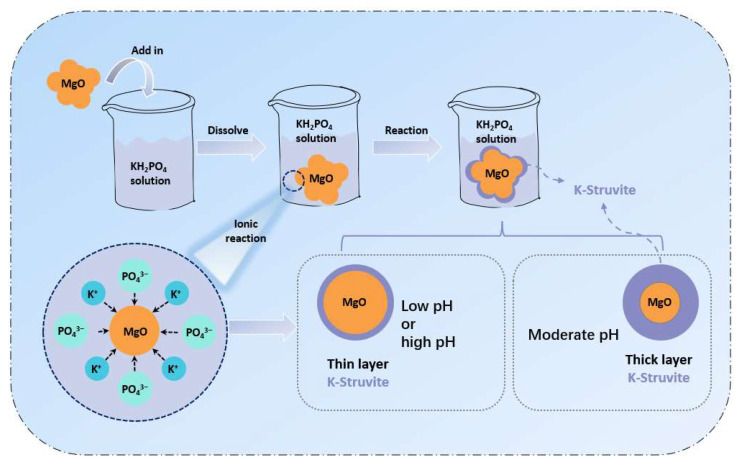
Possible effect of pH on K-struvite formation in MKPC systems.

Taking reaction time 1 h for example, too-high pH (>6.8) or too-low pH (<6.1) could have an adverse effect on the formation of K-struvite. Among them, the formation of K-struvite is hard at a low pH due to thermodynamics, while too-high pH will cause a dense K-struvite layer rapidly covering the MgO particles, thus hindering the further formation of K-struvite, as seen in Figure 4. 

In particular, when the Mg/P ratio is 1, the final pH of the solution is about 7.65, which is consistent with the literature [32]. However, in practical engineering applications, when the Mg/P ratio is large, the amount of raw material dead burnt MgO increases, and the content of CaO or other metal oxides in impurities increases, which will affect the final pH value of the solution to some extent, thereby affecting the hydration process of MKPC.

For the analysis of the effect of pH on the type and population of hydration products, details are present in Section 3.5.

### 3.2. Concentrations of Phosphorus and Potassium in Leachate

**Figure 5:**
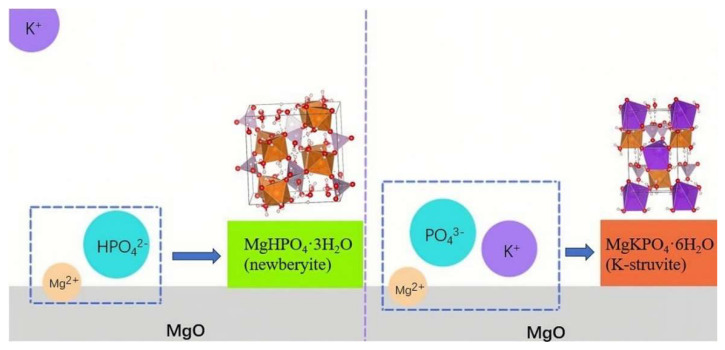
The effect of K^+^ diffusion on the hydration of MKPC. On the left side, the K^+^ diffuses weakly; thus, it is more likely to form newberyite. On the right side, the K^+^ has a high degree of diffusion and could spread smoothly on the MgO surface, so it is easier to form K-struvite.

According to existing research, the hydration mechanism of MKPC is roughly divided into two stages. The first stage is the dissolution of phosphate and MgO, and the second stages is the chemical reaction of Mg^2+^, HPO_4_^2−^ and K^+^ to generate the struvite phase. Most studies focus on the first stage: for example, when developing retarders, they always tends to find a retarder adsorbing on the surface of MgO to delay the dissolution of MgO, such as borax [47], sodium triphosphate [48], citric acid [49] and so on. However, there is little research on the second stage of hydration mechanism. The influence of K^+^ on MKPC is evident. If the diffusion degree of K^+^ is insufficient, Mg^2+^ will react with HPO_4_^2−^ to generate newberyite instead of K-struvite, as shown in Figure 5. This is the focus of this work. The remaining K^+^ in aqueous solution is discussed in this section, as well as the XRD refinement results and MD simulation in Section 3.3 and Section 3.5, are all aimed at understanding the influence of K^+^ diffusion kinetics on the hydration of MKPC.

The concentrations of phosphorus-containing ions were analyzed by ICP-OES. It should be noted that there may be PO_4_^3−^, HPO_4_^2−^ and H_2_PO_4_^−^ in the solution. Since they were in a dynamic equilibrium state in the solution, they are considered as a whole when studying concentrations of phosphorus. The concentration of potassium ion is analyzed by AAS (Atomic Absorption Spectroscopy).

It could be seen from Table 4 and Table 5 that in all the samples, the consumption degrees of phosphorus and potassium were not consistent (both phosphorus and potassium were derived from KH_2_PO_4_, and the initial molar ratio of phosphorus and potassium is 1:1, shown in Table 5), and phosphorus is more easily involved in crystal phase formation and consumed than potassium. The Mg/P ratios have a significant effect on the consumption of P and K. When the Mg/P ratio is 1:1, 61.52% of P is involved in the reaction, while only 12.72% of K is involved, which is not conducive to the formation of K-struvite crystal phase. A higher Mg/P ratio does not lead to a lower amount of K^+^ and P-containing acid radical ions in solution. The content of K^+^ and P-containing acid ions in solution with Mg/P ratio of 7:1 is even higher than that of solution with Mg/P ratio of 3:1. Generally speaking, MP3 and MP5 had better property for the consumption of P and K. The Mg/P ratios have a significant and relatively complex effect on the hydration process of MKPC. In addition, the content of phosphorus in solution is less than that of potassium, which is probably due to the formation of solid phosphate without potassium in each group of samples.

### 3.3. Rietveld XRD Refinement

Since the reaction of KH_2_PO_4_ was always incomplete in Equation (1), it was intuitive that increasing the Mg/P ratio will increase the reaction of KH_2_PO_4_ and thus increase K-struvite production. However, due to the presence of by-products, this trend will change as the Mg/P ratio increases to a certain extent. This will be further discussed in Section 3.5.

**Figure 6:**
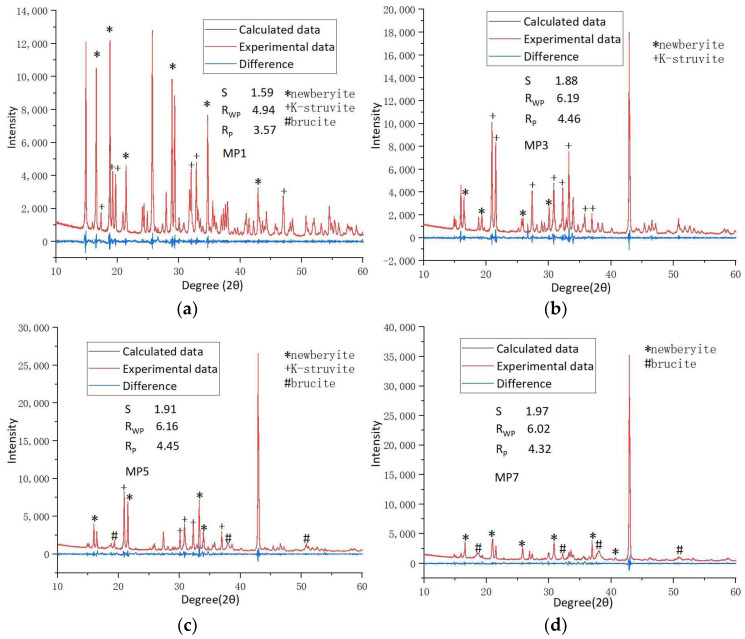
X-ray diffraction patterns for (**a**) MP1, (**b**) MP3, (**c**) MP5, (**d**) MP7.

**Figure 7:**
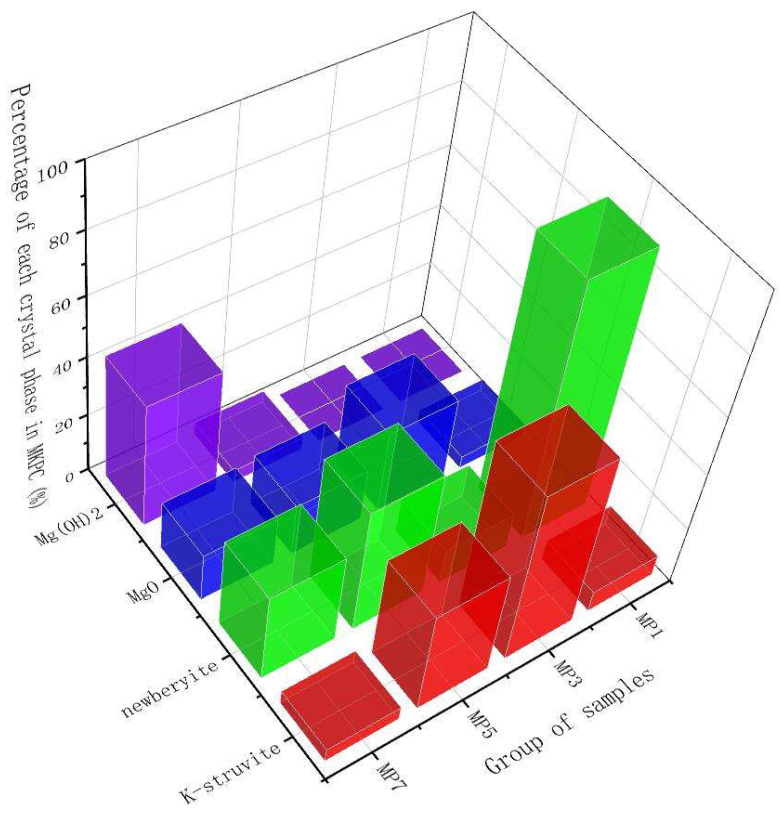
Rietveld XRD refinement: mineral phase population (%) of hydration products of MKPC.

In order to quantitatively study the type and distribution of hydration products of MKPC under different Mg/P, the samples were analyzed by Rietveld XRD refinement (shown in Figure 6). S, R_wp_, and R_p_ values are all parameters to evaluate if the Rietveld XRD refinement is credible. S means goodness-of-fit. R_wp_ means R-weighted pattern. R_p_ means R-pattern. The peak signals of 26 degrees and 43 degrees came from the unreacted MgO crystal in the raw material, which is not marked in the figure because it is not a hydration product of the MKPC system. Figure 7 shows the mineral phase distribution of hydration products of MKPC. When the Mg/P ratio was 1:1, the most important factor is pH value. Due to the insufficient alkaline environment of the solution, newberyite was the main product. However, when the Mg/P ratio was higher than 3:1, the population of K-struvite crystal phases (or even the amount of K-struvite crystal phases) decreased gradually with the increase in Mg/P ratios unexpectedly (shown in Figure 7). The reason may be that the decisive step in the formation of K-struvite crystal phase is the cementation speed of K^+^, that is, after the infiltration and hydration of MgO, the utilization rate of PO_4_^3−^ and K^+^ is not consistent, with PO_4_^3−^ faster and K^+^ slower (shown in Table 4). Therefore, when the Mg/P ratio gradually increased, the reaction rate of K^+^ was not enough to form more K-struvite, and the PO_4_^3−^ turn to form newberyite and brucite phase. This is verified by the heterogeneous nucleation mechanism for the K-struvite crystals [29]: after the hydration of MgO, Mg(H_2_O)_6_^2+^ attracts negatively charged PO_4_^3−^ and finally positively charged K^+^ or H^+^. There is competition between K^+^ and H^+^. When Mg/P ratio increased to 3:1, the main transformation mineral phase of MgO was K-struvite. When Mg/P ratio increased to 5:1, K^+^ reactivity was low and relatively insufficient, the formation of K-struvite was reduced and the main transformation mineral phase of MgO was newberyite. When Mg/P increased to 7:1, the activities of K^+^ and PO_4_^3−^ were relatively low, and MgO was directly converted to brucite.

### 3.4. SEM and EDS Analysis

**Figure 8:**
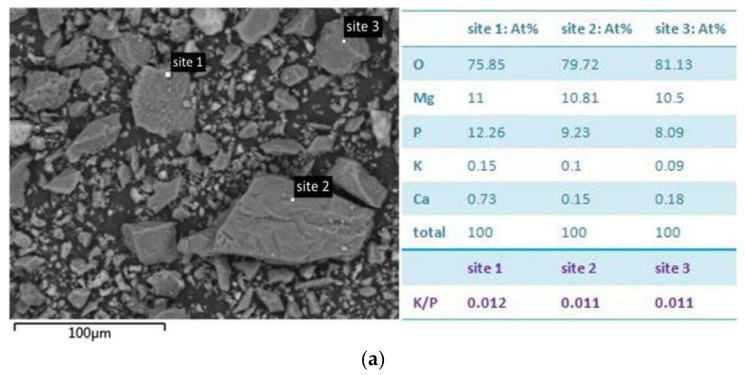

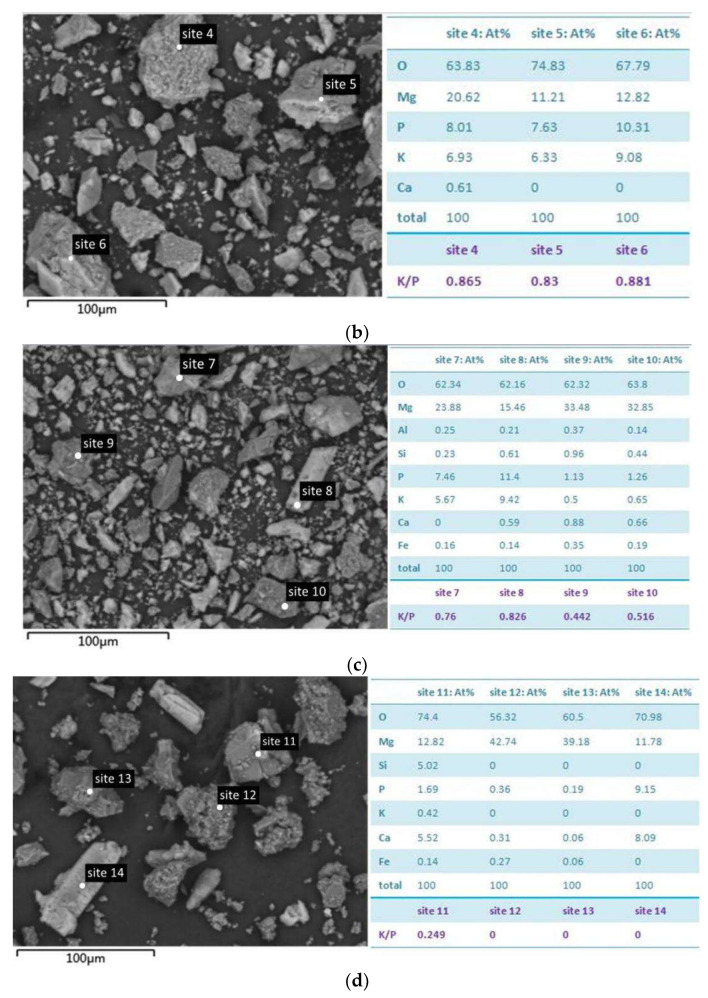
SEM and EDS analyses of MKPC hydration products for (**a**) MP1, (**b**) MP3, (**c**) MP5, (**d**) MP7.

To understand the effect of different Mg/P ratio on the hydration products of MKPC, SEM and EDS were used to observe the microscopic morphology and element distribution of the samples (shown in Figure 8).

Here, the potassium-to-phosphorus ratio (the potassium-to-phosphorus ratio in this paragraph refers to the ratio of the amounts of substances), K/P, is a relatively stable indicator to identify the K-struvite. In pure K-struvite crystal phase (MgKPO_4_·6H_2_O), the K/P ratio is 1. For sample MP1, the K/P of site 1, site 2 and site 3 on the blocky sample were all far lower than 1, indicating that the content of K-struvite crystal phase is extremely low, and the main component of the sample was blocky newberyite. For the sample MP3, the K/P of site 4, site 5 and site 6 in the blocky sample were all close to 1, indicating that the main crystal phase in the sample of MP3 was K-struvite. For sample MP5, the K/P of site 7 on the bulk solid and site 8 on the bar-shaped solid were close to 1, and these crystalline phases were mainly K-struvite. The K/P of sites 9 and 10 on the blocky solid were close to 0.5, indicating that some of these samples were K-struvite, and the other parts were presumed to be dominated by MgO and brucite. For sample MP7, the K/P of site 11, site 12 and site 13 on the blocky solid were much lower than 1, mainly MgO and brucite. The K/P of site 14, a small amount of bar-shaped solid, was close to 1, and these crystalline phases were mainly K-struvite. In general, the pictures of SEM and EDS show that the ratio of magnesium to phosphorus has a significant effect on the type and distribution of hydration products of MKPC. According to different ratios of Mg/P, from M/P = 1:1 to M/P = 7:1, the dominant hydration product could be newberyite, K-struvite, newberyite (with quite a few K-struvite) and brucite, respectively.

### 3.5. Analysis of Molecular Dynamics Simulation Calculations

Here, this work proposed a “transition zone theory” to describe the hydration process of MKPC. That is, the hydration process of MKPC was not only an acid–base reaction but also a solid/liquid interface reaction. Thus, MKPC system could be divided into three zones from the solid to the liquid: solid matrix, transition zone and liquid zone. There were two preconditions about the crystallization of K-struvite. One was that K^+^ should diffuse from liquid zone to transition zone to meet with Mg^2+^. Otherwise, newberyite (MgHPO_4_·3H_2_O), cattite (Mg_3_(PO_4_)_2_·22H_2_O), phosphorrösslerite (MgHPO_4_·7H_2_O) or Mg_2_KH(PO_4_)_2_·15H_2_O would be the main hydration products of MKPC system due to the lack of K^+^. The other precondition is that K^+^ and Mg^2+^ should be located on the surface of MgO in order to facilitate the heterogeneous nucleation process of K-struvite crystal. Obviously, in both of these two processes, the diffusion capacity of K^+^ played a dominant role.

**Figure 9:**
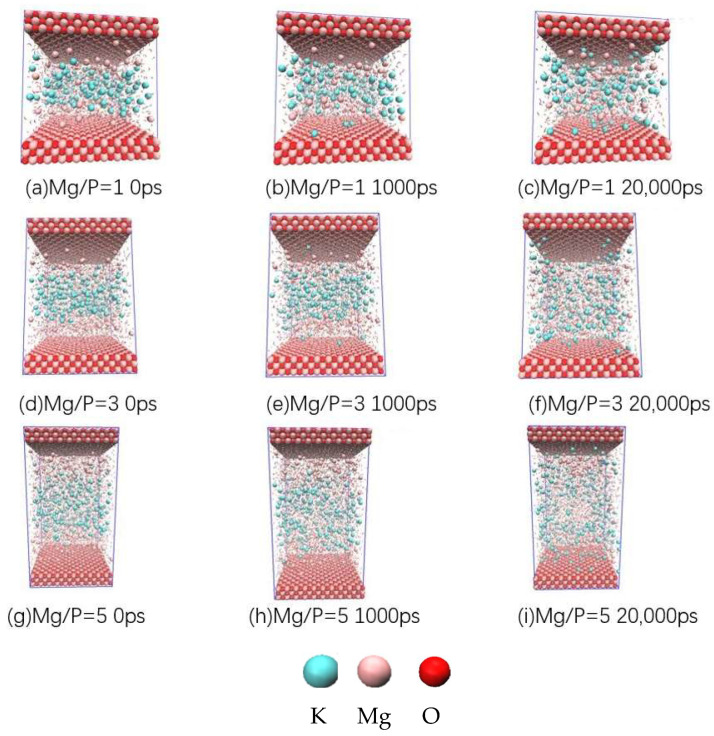
Flash pictures of MD simulation process. Please refer to the movie attachment for detailed video animation.

**Figure 10:**
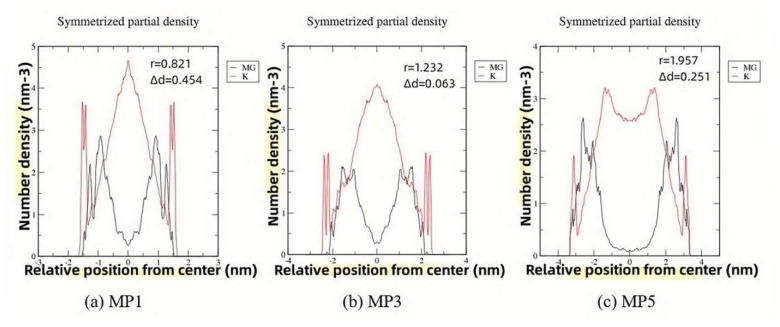
The number density distribution charts with Mg/P of 1, 3, 5.

Specifically, the spatial distribution of K^+^ and Mg^2+^ in the system was the primary concern of this work, as it was a prelude to determining which mineral phases will be formed in the system. Flash pictures of MD simulation process was shown in Figure 9. Different Mg/P would obviously offer different upfront conditions. Here were two focuses of attention. One was that when Mg^2+^ diffused from the surface of the MgO phase to the solution and met the K^+^ diffused from the solution, according to the stoichiometric ratio of Equation (1), the 1:1 ratio of Mg^2+^ and K^+^ was suitable to generate K-struvite. This distance (locating at the first cross point of red line and black line in Figure 10) from the central plane was defined as r. The larger the r was, the stronger diffusion ability of K^+^ was, and the diffusion of K^+^ was the decisive step in the formation of K-struvite in most cases. Therefore, a larger r value meant a tendency towards the generation of K-struvite. Second, the formation of K-struvite mineral phases tends to trigger on the surface of the undissolved MgO solid phase, so the distance from the nearest K^+^ to the MgO surface was defined as Δd. The smaller the Δd was, the more favorable the formation of K-struvite was. Compared with other non-K-struvite mineral phases in these systems, such as newberyite, because they did not contain K^+^, the larger the Δd was, the easier the formation of non-K-struvite mineral phase was, without K-struvite competition. 

Figure 10 shows the number density distribution along the Z axis of K^+^ and Mg^2+^ after 20 ns molecular dynamics simulation with Mg/P of 1, 3, 5, respectively. When Mg/P was 1, a small r value indicated a slow diffusion rate of K^+^, while a large Δd value greatly increased the formation of hydration products without K, newberyite, which was consistent with the results of XRD refinement. When Mg/P was 3, a large r value indicated a faster diffusion rate of K^+^, while a small Δd avoided the formation of newberyite in favor of K-struvite. When Mg/P was 5, a large r value indicated a fast diffusion rate of K^+^, while a moderate Δd value resulted in the formation of both K-struvite and newberyite. These results were also consistent with the results of XRD refinement.

At the same time, part of the K^+^ ions, which could not participate in the K-struvite reaction, existed in the aqueous solution. AAS characterization results (seen in Table 3 and Table 4), XRD refinement (seen in Table 5) and MD simulations confirmed each other, revealing the mechanism of MKPC hydration process. Different Mg/P values could induce different K^+^ diffusion effect, which in turn affected the type, quantity and proportion of hydration products of MKPC.

### 3.6. Effects of K^+^ Diffusion with Different Mg/P on the Conversion Path of MgO

As could be seen from the experimental results and MD simulation analyses in the previous sections, K^+^ diffusion with different Mg/P ratios had an important influence on the hydration mechanism of MKPC. 

**Figure 11:**
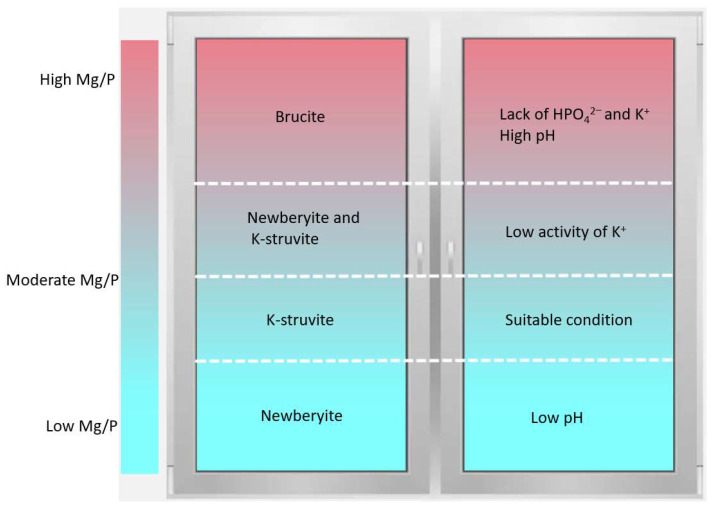
Four parts model: Mg/P, principal crystalline phases and decisive factors.

**Figure 12:**
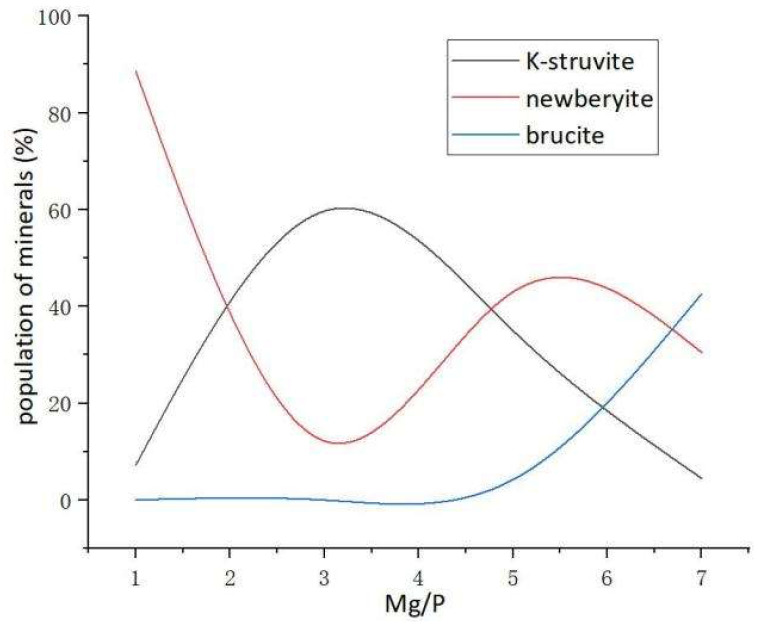
The function between Mg/P and population of minerals (%).

By affecting the diffusion kinetic of K^+^, Mg/P ratios controlled the type of hydration products of MKPC and their proportion as well. Here, we give the figure of Mg/P, principal crystalline phases and decisive factors in Figure 11. For clarifying the influence of Mg/P ratio on the hydration mechanism of MKPC, the function between Mg/P and population of minerals (%) is shown in Figure 12. When the Mg/P ratio increased from 1:1 to 7:1, the mineral phases of hydration products went through four parts of newberyite, K-struvite, newberyite (with quite a few K-struvite) and brucite, as shown in Figure 11. When the Mg/P ratio is 1:1, because the total amount of MgO is low, it could not provide enough alkaline environment, so the hydration products are dominated by newberyite. When the Mg/P ratio increases to 3:1, the proportion of MgO, PO_4_^3−^ and K^+^ was appropriate and the pH of the solution was suitable, and thus K-struvite dominated the hydration products of MKPC in this case. With further increase in MgO content in the system till a Mg/P ratio of 5:1, the further formation of K-struvite was limited due to the insufficient K^+^ concentration, and the excessive MgO and HPO_4_^2−^ could only transform to newberyite. In other words, due to the inactive chemical kinetics of K^+^, additional MgO could not accelerate the formation of K-struvite, but instead reduced the amount of K-struvite, so the hydration products are dominated by newberyite. When the Mg/P ratio rises to 7:1, the formations of K-struvite and newberyite are restricted due to the relatively insufficient content of K^+^ and PO_4_^3−^. At the same time, high pH would also restrain the formation of K-struvite. The most preferred hydration product is brucite in this case, and the formation of brucite inhibited the formation of K-struvite and newberyite. That is, due to the chemical kinetics of K^+^ and PO_4_^3−^, more MgO could not accelerate the formation of K-struvite (and newberyite), but instead reduced the production of K-struvite (and newberyite), so the hydration products are dominated by brucite.

K-struvite is the main cementing phase of MKPC and should be generated as much as possible. Both newberyite and brucite are dangerous to the potential cracking and expansion of MKPC and should be avoided as much as possible. Different Mg/P ratios could adjust the population of MKPC hydration products to provide a better cementing performance.

## 4. Conclusions

In this study, dead burnt MgO and KH_2_PO_4_ were mixed under the condition of w/s = 10 and cured for 21 days to study the formation mechanisms of K-struvite and newberyite under different Mg/P ratios in MKPC. Corresponding MD simulation work was used to explain the hydration mechanism of MKPC. The distribution of crystals of MKPC could be designed by different Mg/P ratios. The main conclusions of this paper are drawn as follows: 

The mechanism of the effect of Mg/P on the hydration products of MKPC is that Mg/P affects the diffusion degree of K^+^ in the MKPC system, and the system which lacks K^+^ on the MgO surface would produce a large amount of newberyite instead of K-struvite. Large amounts of newberyite lead to a potential cracking risk for MKPC systems.

With the gradual increase in Mg/P ratios, the main hydration products of MKPC would go through different parts. When the Mg/P ratios are 1:1, 3:1, 5:1 and 7:1, the dominant crystalline phases in the hydration products of MKPC are newberyite, K-struvite, newberyite (with quite a few K-struvite) and brucite, respectively. The crystalline phase distribution of MKPC could be adjusted as a function of different Mg/P ratios.

The reason for the existence of the function relationship is that the reactivity of K^+^ is lower than that of PO_4_^3−^ during the hydration of MKPC, and the formation of brucite would inhibit the formation of K-struvite (and newberyite). Low pH leads to formation of newberyite and high pH restrains the formation of K-struvite, and suitable pH would be beneficial to the formation of K-struvite. In this context, the appropriate Mg/P ratio for MKPC should be between 3:1 and 5:1, not too high or too low.

However, this work was carried out under the condition of w/s = 10. The effect of Mg/P ratios on MKPC hydration products under the condition of low w/s should be further studied. 

## Figures and Tables

**Table 1:** XRF oxide analysis of dead burnt MgO, precision ± 0.001 wt%.

Oxide	wt%
MgO	85.592
SiO_2_	5.221
CaO	3.014
Fe_2_O_3_	1.824
Al_2_O_3_	1.239
SO_3_	0.251
Na_2_O	0.182
P_2_O_5_	0.138
MnO	0.113
Cr_2_O_3_	0.067
K_2_O	0.047
TiO_2_	0.037
Cl	0.017
SrO	0.007
CO_2_	2.251

**Table 2:** Mix design of the prepared sample (ω), w/s = 10.

	MgO	KH_2_PO_4_	w/s
MP1	0.5	0.5	10
MP3	0.75	0.25	
MP5	0.83	0.17	
MP7	0.875	0.125	

**Table 3:** Initial distribution of ions and molecules in MD models.

Mg/P Ratio	0–4.21 Å	5–25 Å	25–45 Å
1:1	Solid matrix MgO containing 1200 Mg atoms and 1200 O atoms	2227 H_2_O	
		80 Mg^2+^	170 K^+^
		80 HPO_4_^2−^	80 HPO_4_^2−^
		\	10 H_2_PO_4_^−^
3:1	Solid matrix MgO containing 1200 Mg atoms and 1200 O atoms	3245 H_2_O	
		100 Mg^2+^	230 K^+^
		100 HPO_4_^2−^	100 HPO_4_^2−^
		\	30 H_2_PO_4_^−^
5:1	Solid matrix MgO containing 1200 Mg atoms and 1200 O atoms	4200 H_2_O	
		120 Mg^2+^	280 K^+^
		120 HPO_4_^2−^	120 HPO_4_^2−^
		\	34 H_2_PO_4_^−^

**Table 4:** The ion concentrations of MKPC solutions in each group (Unit: M). c_Pi_ and c_Ki_ mean the concentrations of **i**nitial phosphorus-containing ions and potassium ions. c_Pf_ and c_Kf_ mean the concentrations of **f**inal phosphorus and potassium ions. P_l_ (%) and K_l_ (%) mean the percentage of phosphorus and potassium in **l**iquid of the total (unreacted). P_s_ (%) and K_s_ (%) mean the percentage of phosphorus and potassium in **s**olid phase (reacted).

	c_Pi_	c_Pf_	P_l_ (%)	P_s_ (%)	c_Ki_	c_Kf_	K_l_ (%)	K_s_ (%)
MP1	0.567	0.218	38.48	61.52	0.567	0.495	87.28	12.72
MP3	0.389	0.085	21.92	78.08	0.389	0.179	46	54
MP5	0.296	0.021	6.48	93.52	0.296	0.088	29.68	70.32
MP7	0.239	0.058	24.08	75.92	0.239	0.124	52	48

**Table 5:** The amounts of ionic substances (unit: mol) of MKPC solutions in each group. n_Pi_ and n_Ki_ mean the amounts of substances of initial phosphorus-containing ions and potassium ions. n_Pf_ and n_Kf_ mean the amounts of substances of final phosphorus and potassium ion.

	n_Pi_	n_Pf_	n_Ki_	n_Kf_
MP1	0.125	0.0481	0.125	0.1091
MP3	0.125	0.0274	0.125	0.0575
MP5	0.125	0.0081	0.125	0.0371
MP7	0.125	0.0301	0.125	0.0650

## Data Availability

Data are contained within the article.
